# Some monotonicity properties and inequalities for the generalized digamma and polygamma functions

**DOI:** 10.1186/s13660-018-1844-2

**Published:** 2018-09-20

**Authors:** Li Yin, Li-Guo Huang, Zhi-Min Song, Xiang Kai Dou

**Affiliations:** 1 College of Science, Binzhou University, Binzhou City, China; 2 Binzhou Middle School, Binzhou City, China

**Keywords:** 33B15, 26A48, 26A51, Generalized digamma and polygamma functions, Monotonicity, Inequalities, Concavity

## Abstract

Several monotonicity and concavity results related to the generalized digamma and polygamma functions are presented. This extends and generalizes the main results of Qi and Guo and others.

## Introduction

The Euler gamma function is defined for all positive real numbers *x* by

$$ \Gamma(x)= \int_{0}^{\infty}t^{x-1}e^{-t}\,dt. $$

The logarithmic derivative of $\Gamma(x)$ is called the psi or digamma function. That is,

$$ \psi(x)=\frac{d}{dx}\ln\Gamma(x)=\frac{\Gamma'(x)}{\Gamma (x)}=-\gamma - \frac{1}{x}+\sum_{n=1}^{\infty} \frac{x}{n(n+x)}, $$

where $\gamma=0.5772\ldots$ is the Euler–Mascheroni constant, and $\psi^{(m)}(x)$ for $m\in\mathbb{N}$ are known as the polygamma functions. The gamma, digamma and polygamma functions play an important role in the theory of special functions, and have many applications in other many branches, such as statistics, fractional differential equations, mathematical physics and theory of infinite series. The reader may see the references [9–13, 18–20, 24, 45–47, 49]. Some of the work on the complete monotonicity, convexity and concavity, and inequalities of these special functions can be found in [1–6, 8, 14–17, 21, 22, 27–30, 37–42] and the references therein.

In 2007, Diaz and Pariguan [11] defined the *k*-analogue of the gamma function for $k>0$ and $x>0$ as

$$ \Gamma_{k}(x)= \int_{0}^{\infty}t^{x-1}e^{-\frac{t^{k}}{k}}\,dt= \lim_{n\rightarrow\infty}\frac{n!k^{n}(nk)^{\frac{x}{k}-1}}{x(x+k)\cdots (x+(n-1)k)}, $$

where $\lim_{k\rightarrow1}\Gamma_{k}(x)=\Gamma(x)$. Similarly, we may define the *k*-analogue of the digamma and polygamma functions as

$$\psi_{k}(x)=\frac{d}{dx}\ln\Gamma_{k}(x) \quad \mbox{and} \quad \psi _{k}^{(m)}(x)=\frac {d^{m}}{dx^{m}} \psi_{k}(x). $$

It is well known that the *k*-analogues of the digamma and polygamma functions satisfy the following recursive formula and series identities (see [11]):

1.1 $$\begin{aligned}& \Gamma_{k}(x+k)=x\Gamma_{k}(x),\quad x>0, \end{aligned}$$

1.2 $$\begin{aligned}& \psi_{k}(x)=\frac{\ln k-\gamma}{k}-\frac{1}{x}+\sum _{n=1}^{\infty }\frac{x}{nk(nk+x)}, \end{aligned}$$

and

1.3 $$ \psi_{k}^{(m)}(x)=(-1)^{m+1}m!\sum _{n=0}^{\infty}\frac{1}{(nk+x)^{m+1}}. $$

Very recently, Nantomah, Prempeh and Twum [35] introduced a $(p,k)$-analogue of the gamma and digamma functions defined for $p\in\mathbb{N}$, $k>0$ and $x>0$ as

1.4 $$\begin{aligned}& \Gamma_{p,k}(x)= \int_{0}^{p} t^{x-1} \biggl(1- \frac{t^{k}}{pk} \biggr)^{p}\,dt=\frac {(p+1)!k^{p+1}(pk)^{\frac{x}{k}-1}}{x(x+k)\cdots(x+pk)}, \end{aligned}$$

1.5 $$\begin{aligned}& \psi_{p,k}(x)=\frac{d}{dx}\ln\Gamma_{p,k}(x)= \frac{1}{k}\ln (pk)-\sum_{n=0}^{p} \frac{1}{nk+x}, \end{aligned}$$

and

1.6 $$\begin{aligned} \psi_{p,k}^{(m)} (x) =& ( - 1){}^{m}m!\sum _{n = 0}^{p} {\frac{1}{{(nk + x)^{m + 1} }}} \\ =& ( - 1)^{m + 1} \int_{0}^{\infty}{\frac{{1 - e^{ - k(p + 1)t} }}{{1 - e^{ - kt} }}} t^{m} e^{ - xt} \,dt. \end{aligned}$$

It is obvious that $\lim_{p\rightarrow+\infty}\psi_{p,k}(x)=\psi_{k}(x)$. Some important identities and inequalities involving these functions may be found in [30, 34, 35].

In [4], the function $\phi(x)=\psi(x)+\ln(e^{\frac {1}{x}}-1)$ was proved to be strictly increasing on $(0,\infty)$. In [6], it is demonstrated that if $a\leq-\gamma$ and $b\geq0$, then

1.7 $$ a-\ln\bigl(e^{\frac{1}{x}}-1\bigr)< \psi(x)< b-\ln \bigl(e^{\frac{1}{x}}-1\bigr). $$

Furthermore, Guo and Qi [14] showed that the function $\phi(x)$ is strictly increasing and concave on $(0,\infty)$. Attracted by this work, it is natural to look for an extension of (1.7) involving $\psi_{k}(x)$ and $\psi_{p,k}(x)$. On the other hand, Nielsen’s *β*-function has been deeply researched in the last years. In particular, K. Nantomah gave some results on convexity and monotonicity of the function in [31], and obtained some convexity and monotonicity results as well as inequalities involving a generalized form of the Wallis’s cosine formula in [32]. The function can be used to calculate some integrals (see [7, 36]). Recently, K. Nantomah studied the properties and inequalities of a *p*-generalization of the Nielsen’s function in [33]. In this paper, we shall give double inequalities for the *k*-generalization of the Nielsen *β*-function. In addition, it is worth noting that Krasniqi, Mansour, and Shabani presented some inequalities for *q*-polygamma functions and *q*-Riemann Zeta functions by using a *q*-analogue of Hölder type inequality in [23].

The first aim of this paper is to present a new monotonicity theorem for $\psi_{k}(x)$, and give three different proofs. The second aim is to show an inequality for the ratio of the generalized polygamma functions by generalizing a method of Mehrez and Sitnik. The classical Mehrez and Sitnik’s method may be found in [25, 26, 43]. Finally, we also give a new inequality for the inverse of the generalized digamma function.

Our main results read as follows.

### Theorem 1.1

*For* $0< k\leq1$, *the function* $\phi_{k}(x)=\psi_{k}(x)+\ln(e^{\frac {1}{x}}-1)$ *is strictly increasing on* $(0,\infty)$. *In particular*, *the inequalities*

1.8 $$ \frac{\ln k-\gamma}{k}< \psi_{k}(x)+\ln\bigl(e^{\frac{1}{x}}-1 \bigr)< 0 $$

*hold true for* $0< k\leq1$ *and* $x\in(0,\infty)$ *where the constants* $\frac{\ln k-\gamma}{k}$ *and* 0 *in* (1.8) *are the best possible*.

### Remark 1.1

Here, we give an application of Theorem 1.1. Define the *k*-generalization of the Nielsen’s *β*-function as

$$\begin{aligned} \beta_{k}(x)&= \int_{0}^{1} \frac{t^{x-1}}{1+t^{k}}\,dt \\ &= \int_{0}^{\infty} \frac{e^{-xt}}{1+e^{-kt}}\,dt \\ &=\sum^{\infty}_{n=0} \biggl( \frac{1}{2nk+x}-\frac{1}{2nk+k+x} \biggr) \\ &=\frac{1}{2} \biggl\{ \psi_{k} \biggl(\frac{x+k}{2} \biggr)-\psi_{k} \biggl(\frac{x}{2} \biggr) \biggr\} . \end{aligned}$$

By using (1.8), we easily obtain double inequalities of the generalized Nielsen’s *β*-function for $0< k\leq1$ and $x\in (0,\infty)$:

$$\frac{1}{2}\ln \biggl( {\frac{{e^{2/x} - 1}}{{e^{2/(x + k)} - 1}}} \biggr) + \frac{{\ln k - \gamma}}{2k} < \beta_{k} (x) < \frac{1}{2}\ln \biggl( {\frac{{e^{2/x} - 1}}{{e^{2/(x + k)} - 1}}} \biggr) - \frac{{\ln k - \gamma}}{2k}. $$

### Theorem 1.2

*For* $0< k\leq1$, *the function* $\phi_{k}(x)$ *is strictly concave on* $(0,\infty)$. *As a result*, *for* $0< k\leq1$ *and* $x,y\in(0,\infty)$, *we have*

1.9 $$ 2\psi_{k} \biggl(\frac{x+y}{2} \biggr)- \psi_{k}(x)-\psi_{k}(y)\geq\ln \frac {(e^{\frac{1}{x}}-1)(e^{\frac{1}{y}}-1)}{(e^{\frac{2}{x+y}}-1)^{2}}. $$

Using the Theorems 1.1 and 1.2, we easily obtain the following Corollary 1.1.

### Corollary 1.1

*For* $0< k\leq1$ *and* $x\in(0,\infty)$, *we have*

1.10 $$ \psi_{k}'(x)>\frac{1}{(1-e^{-\frac{1}{x}})x^{2}} $$

*and*

1.11 $$ \psi_{k}''(x)< \frac{e^{-\frac{1}{x}}-2x(1-e^{-\frac {1}{x}})}{(1-e^{-\frac {1}{x}})^{2}x^{4}}. $$

### Theorem 1.3

*For* $x>0$ *and* $k\geq1$, *we have*

1.12 $$ \frac{\ln k-\gamma}{k}+x\psi_{k}' \biggl(k+ \frac{x}{2} \biggr)< \psi _{k}(x+k)< \frac{\ln k-\gamma}{k}+x \psi_{k}'\bigl(\sqrt{k(k+x)} \bigr). $$

### Theorem 1.4

*For* $p,k>0$ *and every positive integer* $m\geq4$, *the function*

$$\phi_{m,p,k} (x) = \frac{{ [ {\psi_{p,k}^{(m)} (x)} ]^{4} }}{{\psi_{p,k}^{(m - 3)} (x)\psi_{p,k}^{(m - 1)} (x)\psi_{p,k}^{(m + 1)} (x) \psi_{p,k}^{(m +3)} (x)}} $$

*is strictly decreasing on* $(0,\infty)$ *with*

1.13 $$ \lim_{x \to\infty}\phi_{m,p,k} (x) = \frac {(m-3)(m-2)(m-1)^{2}}{{m^{2} (m+1)(m+2)}} $$

*and*

1.14 $$ \lim_{x \to0}\phi_{m,p,k} (x) = \frac{(m-2)(m-1) m^{2}}{{(m + 1)^{2}(m+2)(m+3)}}. $$

*As a result*, *for* $p,k,x>0$ *and every positive integer* $m\geq4$, *we have*

$$\begin{aligned} \frac{(m-3)(m-2)(m-1)^{2}}{{m^{2} (m+1)(m+2)}} &< \frac{{ [ {\psi_{p,k}^{(m)} (x)} ]^{4} }}{{\psi_{p,k}^{(m - 3)} (x)\psi_{p,k}^{(m - 1)} (x)\psi_{p,k}^{(m + 1)} (x) \psi _{p,k}^{(m +3)} (x)}} \\ &< \frac{(m-2)(m-1) m^{2}}{{(m + 1)^{2}(m+2)(m+3)}}. \end{aligned}$$

### Theorem 1.5

*For* $p,k,x>0$, *the inequalities*

1.15 $$ \frac{k}{{\ln ( {\frac{{B + 2k}}{{B + k}}} )}} < \psi _{p,k}^{ - 1} (x) < \frac{{k(p + 1)e^{kx} }}{{pk - e^{kx} }} + \frac{k}{2} $$

*hold where* $B = \frac{{k(p + 1)e^{kx} }}{{pk - e^{kx} }}$.

## Lemmas

### Lemma 2.1

[42] *If* *f* *is a function defined in an infinite interval* *I* *such that*

$$ f(x)-f(x+\epsilon)>0\quad \textit{and}\quad \lim_{x\rightarrow\infty }f(x)= \delta $$

*for some* $\epsilon>0$, *then* $f(x)>\delta$ *on* *I*.

### Remark 2.1

Lemma 2.1 was first proposed by Professor Feng Qi. It is simple, but has been validated in [15, 41, 42] to be especially effective in proving monotonicity and complete monotonicity of functions involving the gamma, psi and polygamma functions. The reader may refer to [40] and the references therein.

### Lemma 2.2

*For* $k>0$, *the function* $\alpha(x)=[\psi_{k}'(x)]^{2}+\psi_{k}''(x)$ *is positive on* $(0,\infty)$ *if and only if* $k\leq1$.

### Proof

Direct computation yields

$$\begin{aligned} \alpha(x)-\alpha(x+k) &= \bigl[\psi_{k}'(x)- \psi_{k}'(x+k)\bigr] \bigl[\psi_{k}'(x)+ \psi_{k}'(x+k)\bigr]+\psi _{k}''(x)- \psi _{k}''(x+k) \\ &= \frac{2}{x^{2}} \biggl[\psi_{k}'(x)- \frac{1}{2x^{2}}-\frac{1}{x} \biggr] \\ &\triangleq\frac{2}{x^{2}}\beta(x), \end{aligned}$$

and

$$\begin{aligned} \beta(x+k)-\beta(x) &= \psi_{k}'(x+k)- \frac{1}{2(x+k)^{2}}-\frac{1}{x+k}-\psi_{k}'(x)+ \frac {1}{2x^{2}}+\frac{1}{x} \\ &= \frac{1}{x}-\frac{1}{2x^{2}}-\frac{1}{x+k}-\frac{1}{2(x+k)^{2}} \\ &= \frac{2(k-1)x^{2}+2k(k-1)x-k^{2}}{2x^{2}(x+k)^{2}}. \end{aligned}$$

It is easily observed that $\beta(x+k)-\beta(x)<0$ if and only if $k\leq1$. We complete the proof by using Lemma 2.1. □

### Lemma 2.3

*The following limit identity holds true*:

2.1 $$ \lim_{x\rightarrow0^{+}} \biggl[\ln\bigl(e^{\frac{1}{x}}-1 \bigr)-\frac {1}{x} \biggr]=0. $$

### Proof

By applying twice l’Hôspital rule, we easily complete the proof. □

### Lemma 2.4

*For* $k>0$, *the inequalities*

2.2 $$ \frac{1}{kx}\leq\psi_{k}'(x)\leq \frac{1}{kx}+\frac{1}{x^{2}} $$

*hold true for any* $x\in(0,\infty)$.

### Proof

Using the inequalities in [34], namely

2.3 $$ \frac{1}{k} \biggl(\frac{1}{x}-\frac{1}{x+pk+k} \biggr)\leq\psi _{p,k}'(x)\leq\frac{1}{k} \biggl( \frac{1}{x}-\frac{1}{x+pk+k} \biggr)+\frac {1}{x^{2}}- \frac{1}{(x+pk+k)^{2}}, $$

we easily obtain (2.2) as $p\rightarrow+\infty$. □

### Lemma 2.5

([25, 26, 43, 48])

*Let* $\{a_{n}\}$ *and* $\{b_{n}\}$ ($n=0,1,2,\ldots$) *be real numbers such that* $b_{n}>0$ *and* $\{\frac{a_{n}}{b_{n}}\}_{n\geq0}$ *be increasing* (*resp*., *decreasing*), *then* $\{\frac{a_{0}+a_{1}+\cdots+a_{n}}{b_{0}+b_{1}+\cdots+b_{n}}\}$ *is increasing* (*resp*., *decreasing*).

### Lemma 2.6

*For* $p,k,x>0$ *and every positive integer* $m\geq2$, *the following limit identity holds true*:

$$\lim_{x\rightarrow0^{+}} x^{m + 1}\psi_{p,k}^{(m)} (x) = \frac{{( - 1)^{m} (m - 1)!}}{k}. $$

### Proof

Considering the inequalities (see [34, Theorem 2.7])

$$\frac{1}{k} \biggl( {\frac{1}{x} - \frac{1}{{x + pk + k}}} \biggr) \le \psi'_{p,k} (x) \le\frac{1}{k} \biggl( { \frac{1}{x} - \frac{1}{{x + pk + k}}} \biggr) + \frac{1}{{x^{2} }} - \frac{1}{{ ( {x + pk + k} )^{2} }} $$

and differentiating them $m-1$ times, we easily complete the proof. □

## Proofs of theorems

### First proof of Theorem 1.1

A simple calculation gives

$$\begin{aligned} e^{\phi_{k}(x)}&=e^{\psi_{k}(x)}\bigl(e^{\frac{1}{x}}-1\bigr)=e^{\psi_{k}(x)+\frac {1}{x}}-e^{\psi_{k}(x)} \\ &=e^{\psi_{k}(x+k)}-e^{\psi_{k}(x)} \\ &\triangleq{\delta_{k}(x)} \end{aligned}$$

and

$$\begin{aligned} \delta_{k}'(x)&=e^{\psi_{k}(x+k)}\psi_{k}'(x)-e^{\psi_{k}(x)} \psi_{k}'(x) \\ &\triangleq\mu_{k}(x+k)-\mu_{k}(x). \end{aligned}$$

Using Lemma 2.2, we easily obtain

$$\mu_{k}'(x)=e^{\psi_{k}(x)}\bigl[\bigl( \psi_{k}'(x)\bigr)^{2}+\psi_{k}''(x) \bigr]>0. $$

This implies that the function $\mu_{k}(x)$ is strictly increasing, and so $\delta_{k}'(x)>0$ on $(0,\infty)$. As a result, the function $e^{\phi _{k}(x)}$ is also strictly increasing on $(0,\infty)$. Considering Lemma 2.3, we have

$$ \lim_{x\rightarrow0^{+}}\phi_{k}(x)=\frac{\ln k-\gamma}{k}\quad \mbox{and}\quad \lim_{x\rightarrow\infty}\phi_{k}(x)=0. $$

The proof of Theorem 1.1 is completed. □

### Second proof of Theorem 1.1

It is easily observed that $\delta_{k}'(x)>0$ is equivalent to

3.1 $$ e^{\frac{1}{x}}\psi_{k}'(x+k)- \psi_{k}'(x)>0. $$

Considering Lemma 2.4, we only need to prove

3.2 $$ e^{\frac{1}{x}}\frac{1}{k(x+k)}>\frac{1}{kx}+ \frac{1}{x^{2}}. $$

Taking the logarithm to both sides of (3.2), we prove

3.3 $$ \frac{1}{x}+\ln\frac{1}{k}+\ln\frac{1}{x+k}>\ln \frac{x+k}{kx^{2}}. $$

So, we only need to prove

3.4 $$ \lambda_{k}(x)=\frac{1}{x}-\ln k-\ln{(x+k)}-\ln \frac{x+k}{kx^{2}}>0. $$

Since $k\leq1$, we easily get

3.5 $$ \lambda_{k}'(x)=\frac{-2kx^{2}+(1-k)x+k(1-k)}{kx^{2}(x+k)}< 0. $$

This implies that the function $\lambda_{k}(x)$ is strictly decreasing on $(0,\infty)$ with $\lim_{x\rightarrow\infty}\lambda_{k}(x)=0$. Hence, we have $\lambda_{k}(x)>0$. The proof is completed. □

### Third proof of Theorem 1.1

Direct calculation results in

3.6 $$ \phi_{k}'(x)=\psi_{k}'(x)- \frac{e^{\frac{1}{x}}}{(e^{\frac{1}{x}}-1)^{2}x^{2}} $$

and

3.7 $$ \phi_{k}'(x)-\phi_{k}'(x+k)= \frac{1}{x^{2}}-\frac{e^{\frac {1}{x}}}{(e^{\frac {1}{x}}-1)x^{2}}+\frac{e^{\frac{1}{x+k}}}{(e^{\frac{1}{x+k}}-1)(x+k)^{2}} $$

with $\lim_{x\rightarrow+\infty}\phi_{k}'(x)=0$.

In order to prove $\phi_{k}'(x)-\phi_{k}'(x+k)>0$ for $x>0$, it suffices to show

3.8 $$ x^{2}\bigl(e^{\frac{1}{x}}-1\bigr)>(x+k)^{2} \bigl(1-e^{-\frac{1}{x+k}} \bigr). $$

So, we only need to prove

3.9 $$ 1-k+\sum_{n=3}^{\infty} \frac{1}{n!} \biggl(\frac{1}{x^{n-2}}+\frac {(-1)^{n}}{(x+k)^{n-2}} \biggr)>0, $$

which is valid. By using Lemma 2.1, we can conclude that $\phi _{k}'(x)>0$. Hence, the function $\phi_{k}(x)$ is strictly increasing on $(0,\infty)$. □

### Proof of Theorem 1.2

Using formula (3.7), we have

$$\begin{aligned} \phi_{k}''(x)-\phi_{k}''(x+k)&= \bigl(\phi_{k}'(x)-\phi_{k}'(x+k) \bigr)' \\ &=\frac{e^{\frac{1}{x+k}} [1+2k+2x-2(x+k)e^{\frac{1}{x+k}} ]}{ (e^{\frac{1}{x+k}}-1 )^{2}(x+k)^{4}} -\frac{(1-2x)e^{\frac{1}{x}}+2x}{(e^{\frac{1}{x}}-1)^{2}x^{4}}. \end{aligned}$$

For $x>0$, the fact $\phi_{k}''(x)-\phi_{k}''(x+k)<0$ is equivalent to

3.10 $$ \frac{(e^{\frac{1}{x}}-1 )^{2}}{(e^{\frac{1}{x+k}}-1 )^{2}}> \frac{(x+k)^{4}}{x^{4}}\frac{(1-2x)e^{\frac{1}{x}}+2x}{e^{\frac {1}{x+k}} [1+2k+2x-2(x+k)e^{\frac{1}{x+k}} ]}. $$

Applying inequality (3.8), we need to prove

3.11 $$ \triangle_{k}(x)=2k+1+2x-2ke^{\frac{1}{x+k}}-(1+2x)e^{\frac {1}{x}+\frac {1}{x+k}}< 0. $$

An easy calculation yields

$$\begin{aligned} \triangle_{k}'(x)&=\frac{ [4(1-k)x^{3}+(2+4k-2k^{2})x^{2}+(2k+2k^{2})x-2x^{4}+k^{2} ]e^{\frac {1}{x}+\frac {1}{x+k}}}{x^{2}(x+k)^{2}} \\ &\quad {}+\frac{2k e^{\frac{1}{x+k}}}{(x+k)^{2}}+2 \end{aligned}$$

and

$$ \triangle_{k}''(x)=\frac{q_{n}(x)e^{\frac{1}{x+k}}+r_{n}(x)e^{\frac {1}{x}+\frac{1}{x+k}}}{x^{4}(x+k)^{4}} $$

with $\lim_{x\rightarrow\infty}\triangle_{k}'(x)=0$, where

$$ q_{n}(x)=-4kx^{5}-2k(1+2k)x^{4} $$

and

$$\begin{aligned} r_{n}(x) =&4(k-3)x^{5}+2\bigl(2k^{2}-13k-2 \bigr)x^{4}-4k(2+7k)x^{3} \\ &{}-8k^{2}(1+2k)x^{2}-4k^{3}(1+k)x-k^{4}. \end{aligned}$$

For $0< k\leq1$, we easily obtain

$$ q_{n}(x)< 0, \qquad r_{n}(x)< 0. $$

This implies that $\triangle_{k}'(x)$ is strictly decreasing and $\triangle_{k}(x)$ is strictly increasing on $(0,\infty)$. Using $\lim_{x\rightarrow\infty}\triangle_{k}(x)=-4<0$ and Lemma 2.1, we complete the proof. □

### Proof of Theorem 1.3

Using (1.1) and (1.2), we get

$$ \psi_{k}(x+k)=\psi_{k}(x)+\frac{1}{x}= \frac{\ln k-\gamma}{k}+\sum_{n=1}^{\infty} \biggl( \frac{1}{nk}-\frac{1}{nk+x} \biggr). $$

By the mean value theorem for differentiation, there exists a number $\sigma_{k,n}=\sigma_{k,n}(x)$ such that $0<\sigma_{k,n}<x$ and

$$ \frac{1}{nk}-\frac{1}{nk+x}=\frac{x}{(nk+\sigma_{k,n})^{2}}. $$

Hence, we find

$$ \sigma_{k,n}=\sqrt{nk(nk+x)} -nk. $$

It is well known that the function $\sigma_{k,n}$ is strictly increasing in *k* on $[1,+\infty)$ with

$$\begin{aligned}& \sigma_{k,1}=\sqrt{k(k+x)} -k, \\& \sigma_{k,\infty}=\lim_{n\rightarrow\infty}\sigma_{k,n}= \frac{x}{2}. \end{aligned}$$

Therefore, we get

$$ x\sum_{n=1}^{\infty}\frac{1}{(nk+\sigma_{k,\infty})^{2}}< \psi _{k}(x+k)-\frac {\ln k-\gamma}{k}< x\sum_{n=1}^{\infty}\frac{1}{(nk+\sigma_{k,1})^{2}}. $$

This completes the proof. □

### Proof of Theorem 1.4

By (1.6) and direct computation, we have

$$\begin{aligned}& \frac{{ ( {\psi_{p,k}^{(m)} (x)} )^{4} }}{{\psi_{p,k}^{(m - 3)} (x)\psi_{p,k}^{(m - 1)} (x)\psi_{p,k}^{(m + 1)} (x)\psi _{p,k}^{(m + 3)} (x)}} \\& \quad = A\frac{{\sum_{n = 0}^{p} {\sum_{\lambda= 0}^{n} {\sum_{k = 0}^{\lambda}{\sum_{i = 0}^{n - \lambda} {\frac{1}{{(ik + x)^{2m + 2} ((n - i)k + x)^{2m + 2} }}} } } } }}{{\sum_{n = 0}^{p} {\sum_{\lambda = 0}^{n} {\sum_{k = 0}^{\lambda}{\sum_{i = 0}^{n - \lambda} {\frac {1}{{(ik + x)^{2m - 2} ((n - i)k + x)^{2m + 4} }}} } } } }}, \end{aligned}$$

where $A = \frac{{ ( {m!} )^{4} }}{{(m - 3)!(m - 1)!(m + 1)!(m + 3)!}}$. Let us define sequences $\{ {\alpha_{m,i} } \}_{i \ge0} $, $\{ {\beta_{m,i} } \}_{i \ge0} $ and $\{ {\omega_{m,i} } \}_{i \ge0} $ by

$$\begin{aligned}& \alpha_{m,i} = \frac{1}{{ ( {ik + x} )^{2m + 2} [ { ( {n - i} )k + x} ]^{2m + 2} }}, \\& \beta_{m,i} = \frac{1}{{ ( {ik + x} )^{2m-2} [ { ( {n - i} )k + x} ]^{2m +4} }}, \end{aligned}$$

and

$$\omega_{m,i} =\frac{{\alpha_{m,i} }}{{\beta_{m,i} }} = \biggl(\frac {{ ( {n - i} )k + x}}{{ik + x}} \biggr)^{4}. $$

It follows that

$$\frac{{\omega_{m,i + 1} }}{{\omega_{m,i} }} = \biggl(\frac{{ [ { ( {n - i - 1} )k + x} ] ( {ik + x} )}}{{ [ { ( {i + 1} )k + x} ] [ { ( {n - i} )k + x} ]}} \biggr)^{4}. $$

It is not difficult to see that the fact $\frac{{\omega_{m,i + 1} }}{{\omega_{m,i} }} < 1 $ is equivalent to

$$\begin{aligned}& \bigl[ { ( {n - i - 1} )k + x} \bigr] ( {ik + x} ) < \bigl[ { ( {i + 1} )k + x} \bigr] \bigl[ { ( {n - i} )k + x} \bigr] \\& \quad \Leftrightarrow\quad - nk^{2} - 2kx < 0. \end{aligned}$$

So the sequence $\{ {\omega_{m,i} } \}_{i \ge0} $ is strictly decreasing. This implies that the function $\phi_{m,p,k} ( x ) $ is strictly decreasing on $(0,\infty)$ by Lemma 2.5. From the identity

$$\psi_{p,k}^{ ( m )} ( {x + k} ) = ( { - 1} )^{m} \frac{{m!}}{{x^{m + 1} }} - ( { - 1} )^{m} \frac {{m!}}{{ ( {x + pk + k} )^{m + 1} }} + \psi_{p,k}^{ ( m )} ( x ), $$

we easily obtain (1.14). Using Lemma 2.6, we get (1.13). This completes the proof. □

### Proof of Theorem 1.5

Using (1.4) and the functional equation (see [35])

$$\Gamma_{p,k} (x + k) = \frac{{pkx}}{{x + pk + k}}\Gamma_{p,k} (x), $$

we obtain, after a direct computation, that

3.12 $$\begin{aligned}& \ln\Gamma_{p,k} (x + k) = \ln(p + 1)! + (p + 1)\ln k + \biggl( {\frac {{x + k}}{k} - 1} \biggr)\ln pk - \sum _{i = 0}^{p} {\ln \bigl( {x + ( {i + 1} )k} \bigr)}, \end{aligned}$$

3.13 $$\begin{aligned}& \ln\Gamma_{p,k} (x) = \ln(p + 1)! + (p + 1)\ln k + \biggl( {\frac {x}{k} - 1} \biggr)\ln pk - \sum _{i = 0}^{p} {\ln ( {x + ik} )}, \end{aligned}$$

and

3.14 $$ \ln\Gamma_{p,k} (x + k) = \ln\frac{{pkx}}{{x + pk + k}} + \ln \Gamma _{p,k} (x). $$

Combining (3.12) and (3.13) with (3.14), we get

3.15 $$ \ln\frac{{pkx}}{{x + pk + k}} = \ln pk - \sum _{i = 0}^{p} {\ln\frac{{x + ( {i + 1} )k}}{{x + ik}}.} $$

By the mean value theorem, we obtain

3.16 $$ \ln\frac{{x + ( {i + 1} )k}}{{x + ik}} = \frac{k}{{ik + \rho(i)}},\quad \rho(i) \in(x,x + k). $$

Hence, identity (3.15) changes into

3.17 $$ \ln\frac{{pkx}}{{x + pk + k}} = k \Biggl( {\frac{1}{k}\ln pk - \sum _{i = 0}^{p} {\ln\frac{1}{{ik + \rho(i)}}} } \Biggr). $$

From identity (3.16), we conclude that

$$\rho(i) = \frac{k}{{\ln ( {1 + \frac{k}{{x + ik}}} )}} - ik. $$

Next, we show that *ρ* is strictly increasing on $(1,\infty)$. Differentiating $\rho(i)$, we observe that $\rho'(i)>0$ if and only if

$$\sqrt{(x + ik) (x + ik + k)} < \frac{{(x + ik + k) - (x + ik)}}{{\ln(x + ik + k) - \ln(x + ik)}}, $$

which follows from the geometric–logarithmic mean inequality. A simple computation yields $\rho(1) = \frac{k}{{\ln ( {\frac{{x + 2k}}{{x + k}}} )}} - k $ and $\rho(\infty) = \lim_{i \to\infty} \rho(i) = x + \frac{k}{2}$. Since $\psi_{p,k}$ and $\psi^{-1}_{p,k}$ are strictly increasing on $(0,\infty)$, we easily obtain that

$$\psi_{p,k} \bigl( {\rho(1)} \bigr) < \frac{1}{k}\ln \frac{{pkx}}{{x + pk + k}} < \psi_{p,k} \bigl( {\rho(\infty)} \bigr). $$

Hence we have

$$\frac{k}{{\ln ( {\frac{{x + 2k}}{{x + k}}} )}} - k < \psi _{p,k}^{ - 1} \biggl( { \frac{1}{k}\ln\frac{{pkx}}{{x + pk + k}}} \biggr) < x + \frac{k}{2}. $$

Replacing *x* by $\frac{{k(p + 1)e^{kx} }}{{pk - e^{kx} }} $ here completes the proof. □

## A conjecture

Finally, we give a conjecture.

### Conjecture 4.1

*For* $p>0$ *and* $k\geq1$, *the function*

$$ \phi_{p,k}(x)=\psi_{p,k}(x)+\ln \bigl(e^{\frac{1}{x}-\frac {1}{x+pk+k}}-1 \bigr) $$

*is strictly decreasing from* $(0,\infty)$ *onto* $(-\infty,\psi_{p,k}(k))$.

### Remark 4.1

It is natural to ask whether the monotonicity result of Theorem 1.1 can be extended to the digamma function $\psi_{p,k}(x)$ with two parameters by using the method of Theorem 1.1. Unfortunately, we failed to prove Conjecture 4.1. Alzer’s work shows that the function $\phi_{k}(x)=\psi_{k}(x)+\ln (e^{\frac {1}{x}}-1)$ is useful for studying harmonic numbers. This is related to the formula (see [35, Remark 2.1])

$$\phi_{p,k}(k)=\frac{1}{k}\bigl[\ln(pk)-H(p+1)\bigr], $$

where $H(n)$ is the *n*th harmonic number. So, it would be a meaningful result if anyone can prove this conjecture.

### Remark 4.2

The $(p,k)$-generalized Nielsen’s *β*-function can be defined as

$$\begin{aligned} \beta_{p,k}(x)&= \int_{0}^{1} \frac{1-t^{2k(p+1)}}{1+t^{k}}t^{x-1}\,dt \\ &= \int_{0}^{\infty} \frac{1-e^{-2k(p+1)t}}{1+e^{-kt}} e^{-xt} \,dt \\ &=\sum^{p}_{n=0} \biggl( \frac{1}{2nk+x}-\frac{1}{2nk+k+x} \biggr) \\ &=\frac{1}{2} \biggl\{ \psi_{p,k} \biggl(\frac{x+k}{2} \biggr)-\psi_{p,k} \biggl(\frac{x}{2} \biggr) \biggr\} , \end{aligned}$$

where $k\in(0,1]$, $p, x \in(0,\infty)$, and $\lim_{p\rightarrow \infty }\beta_{p,k}(x)=\beta_{k}(x)$. Analogously to Remark 1.1, if Conjecture 4.1 holds true, we can estimate the upper and lower bounds of this function $\beta_{p,k}(x)$.

## Results and discussion

Some monotonicity and concavity properties of the *k* and $(p,k)$-analogues of the digamma and polygamma functions were deeply studied. In doing so, we established some inequalities involving the generalized digamma and polygamma functions. Theorems 1.1–1.3 are extensions of some known results. Theorem 1.4 is not only a completely new result, it’s even new for $\psi (x)$. In addition, the method of proof is also new. Theorem 1.5 gives an inequality for the inverse of the digamma function. At the moment, such results are very few. In the end, we stated a conjecture involving the $(p,k)$-analogue of the digamma function.

## Methods and experiment

Not applicable.
